# Correction: Examination of unique volatile organic compound signatures in nasopharyngeal test swab viral transport media using an electronic nose

**DOI:** 10.1371/journal.pone.0339492

**Published:** 2025-12-19

**Authors:** Barbara Swanson, Jessica Bishop-Royse, Ali Keshavarzian, Robert Balk, Abhinav Bhushan, James Moy, Alan Landay, Wrenetha Julion, Jhalak Mehta, Maryan Arrieta, Dylan Behun, Michael Boler, Minnie Kang

The 12^th^ author’s last name is spelled incorrectly. The correct name is: Michael Boler.
